# Supplementary material for: Causal interplay between lactose intolerance and gut microbiota: a combined bidirectional Mendelian randomization and in vivo validation study
Source: Front Nutr. 2026 Jun 1;13:1803337. doi: 10.3389/fnut.2026.1803337 (PMC13265576; doi:10.3389/fnut.2026.1803337)
Supplement: Supplementary file 1 [file Data_Sheet_1.zip › supplementary materials/Forward/forest plot/ebi-a-GCST90027682.finngen_R12_E4_LACTONAS.pdf]

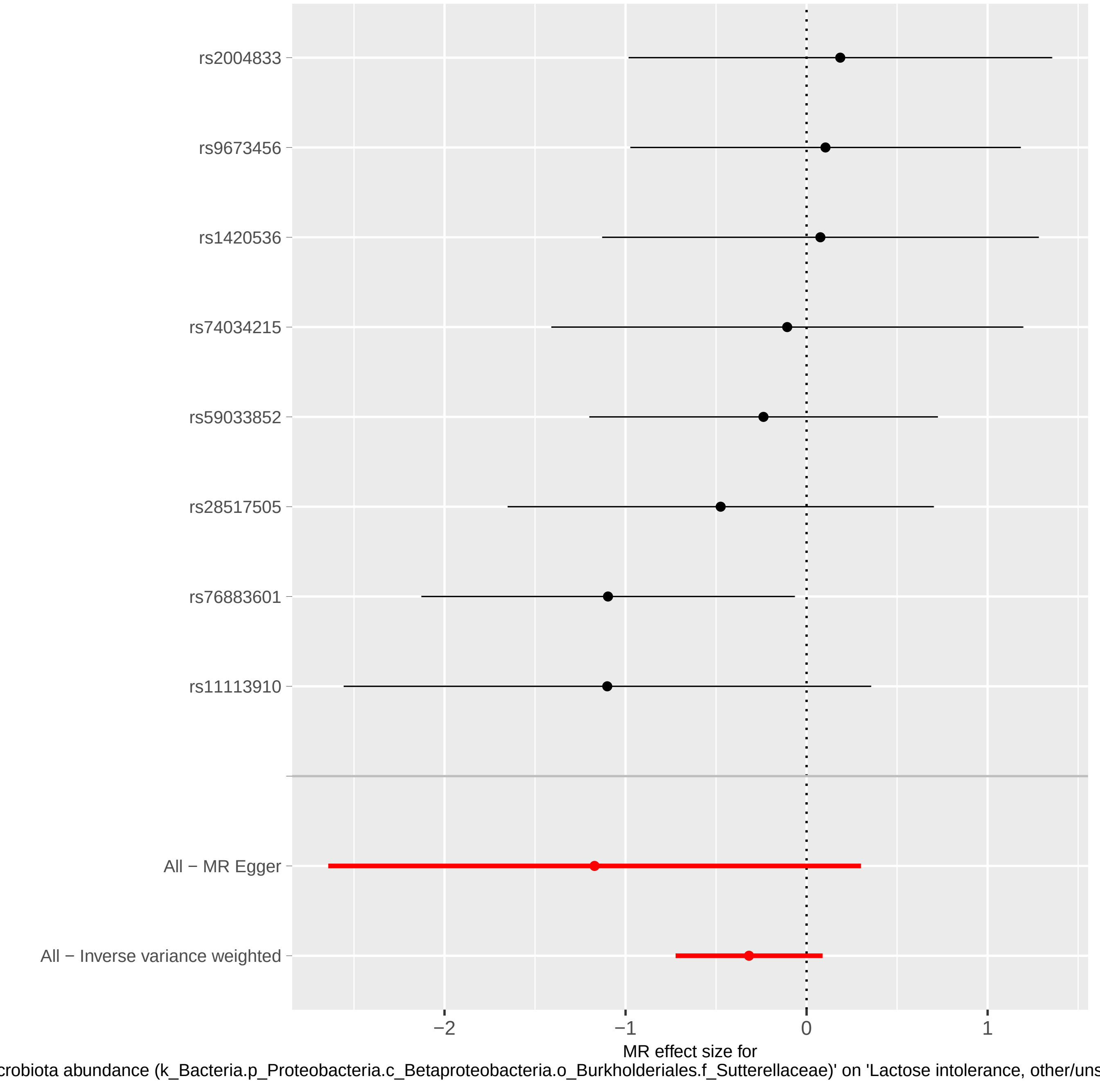
rs2004833
rs9673456
rs1420536
rs74034215
rs59033852
rs28517505
rs76883601
rs11113910
All − MR Egger
All − Inverse variance weighted
−2
−1
0
1
MR effect size for
crobiota abundance (k_Bacteria.p_Proteobacteria.c_Betaproteobacteria.o_Burkholderiales.f_Sutterellaceae)' on 'Lactose intolerance, other/uns
